# Self-reported symptom study of COVID-19 chemosensory dysfunction in Malaysia

**DOI:** 10.1038/s41598-022-06029-6

**Published:** 2022-02-08

**Authors:** Shen-Han Lee, Zhi Xiang Yeoh, Ida Sadja’ah Sachlin, Norzi Gazali, Shahrul Aiman Soelar, Chee Yoong Foo, Lee Lee Low, Sharifah Baizura Syed Alwi, Tengku Mohamed Izam Tengku Kamalden, Jothi Shanmuganathan, Masliza Zaid, Chun Yiing Wong, Hock Hin Chua, Suhaimi Yusuf, Dzawani Muhamad, Philip Rajan Devesahayam, Hong Bee Ker, Zulkiflee Salahuddin, Mahiran Mustafa, Halimuddin Sawali, Heng Gee Lee, Sobani Din, Nor Arisah Misnan, Amran Mohamad, Mohd Noor Ismail, Chenthilnathan Periasamy, Ting Soo Chow, Elang Kumaran Krishnan, Chee Loon Leong, Linda Pei Fang Lim, Nor Zaila Zaidan, Mohd Zambri Ibrahim, Suhaila Abd Wahab, Siti Sabzah Mohd Hashim, Malaysian COVID-19 Anosmia Research Group, Nurul Asyikin Bachok, Linger Sim, Tiana Ti, Teng Huei Lee, Siti Nurul Aliaa Mohd Nor, Kim Siang Tay, Abirami Gouri Pagan, Anura Aman, Kamariah Mohamed Awang, Jamal Nasser Salleh, Harridas Manickam, Nursyamimi Mohamad Zaki, Cheng Keat Moh, Ruthran Thurairaju, Ho Hwee Yee, Nabilah Abd Aziz, Rosdi Ramli, Rosli Mohd Noor, Anilawati Mat Jelani, Mohd Fakri Alimi Mustapha, Abdul Aziez Ab Aziry, Kribananthan Lohanadan, Siti Farhana Abdul Razak, Yen Tsen Saw, Jason Henn Leong Kong, Carren Sui Lin Teh, Kuldip Kaur Prem Singh, Arvindan Karunakaran, Nesha Rajendram, Nik Khairani Nik Mohd, Nurul Amilin Ja’afar, Siti Sarah Che Mohd Razali, Shamesh Baskaran, Farrah Hani Hassan, Kalaiselvi Thuraisingam, Hanisah Hithayathullah, Prempreet Kaur Manjeet Singh, Shen-Han Lee, Nadiah Hanim Zainul, Man Chek Ooi, Siti Aishah Mahadzir, Nurul Afifah Mohd Yusoff, Anees Fatimah Mohamad Illiayas, Yi Shan Tan, Ibtisam Ismail, Huan Keat Chan, Jeyasakthy Saniasiaya, Tiang Koi Ng, Kuganathan Ramasamy, Fatin Farha Mohd Saifuddin

**Affiliations:** 1 Department of Otorhinolaryngology, Hospital Sultanah Bahiyah, KM 6 Jalan Langgar, Alor Setar, 05450 Kedah, Malaysia; 2 Clinical Research Centre, Hospital Sultanah Bahiyah, Kedah, Malaysia; 3 Real World Insights, IQVIA Asia Pacific, Petaling Jaya, Malaysia; 4 Department of Medicine, Hospital Sultanah Bahiyah, Kedah, Malaysia; 5 Department of Otorhinolaryngology, Hospital Sultan Ismail, Johor, Malaysia; 6 Department of Otorhinolaryngology, Hospital Sultanah Aminah, Johor, Malaysia; 7 Department of Medicine, Hospital Sultanah Aminah, Johor, Malaysia; 8 Department of Otorhinolaryngology, Hospital Umum Sarawak, Sarawak, Malaysia; 9 Department of Medicine, Hospital Umum Sarawak, Sarawak, Malaysia; 10 Department of Otorhinolaryngology, Hospital Tengku Ampuan Afzan, Pahang, Malaysia; 11 Department of Medicine, Hospital Tengku Ampuan Afzan, Pahang, Malaysia; 12 Department of Otorhinolaryngology, Hospital Raja Permaisuri Bainun, Perak, Malaysia; 13 Department of Medicine, Hospital Raja Permaisuri Bainun, Perak, Malaysia; 14 Department of Otorhinolaryngology, Hospital Raja Perempuan Zainab II, Kelantan, Malaysia; 15 Department of Medicine, Hospital Raja Perempuan Zainab II, Kelantan, Malaysia; 16 Department of Otorhinolaryngology, Hospital Queen Elizabeth, Sabah, Malaysia; 17 Department of Medicine, Hospital Queen Elizabeth, Sabah, Malaysia; 18 Department of Otorhinolaryngology, Hospital Sungai Buloh, Selangor, Malaysia; 19 Department of Medicine, Hospital Sungai Buloh, Selangor, Malaysia; 20 Department of Otorhinolaryngology, Hospital Sultanah Nur Zahirah, Terengganu, Malaysia; 21 Department of Medicine, Hospital Sultanah Nur Zahirah, Terengganu, Malaysia; 22 Department of Otorhinolaryngology, Hospital Pulau Pinang, Penang, Malaysia; 23 Department of Medicine, Hospital Pulau Pinang, Penang, Malaysia; 24 Department of Otorhinolaryngology, Hospital Kuala Lumpur, Kuala Lumpur, Malaysia; 25 Department of Medicine, Hospital Kuala Lumpur Hospital, Kuala Lumpur, Malaysia; 26 Department of Otorhinolaryngology, Hospital Melaka, Melaka, Malaysia; 27 Department of Medicine, Hospital Melaka, Melaka, Malaysia; 28 Department of Otorhinolaryngology, Hospital Tuanku Fauziah, Perlis, Malaysia; 29 Department of Medicine, Hospital Tuanku Fauziah, Perlis, Malaysia; 30 Hospital Enche’ Besar Hajjah Khalsom, Johor, Malaysia; 31 Department of Otorhinolaryngology, Hospital Tuanku Ja’afar, Negeri Sembilan, Malaysia; 32 Department of Medicine, Hospital Tuanku Ja’afar, Negeri Sembilan, Malaysia

**Keywords:** Viral infection, Epidemiology, Signs and symptoms, Olfactory system

## Abstract

Alterations in the three chemosensory modalities—smell, taste, and chemesthesis—have been implicated in Coronavirus Disease 2019 (COVID-19), yet emerging data suggest a wide geographic and ethnic variation in the prevalence of these symptoms. Studies on chemosensory disorders in COVID-19 have predominantly focused on Caucasian populations whereas Asians remain understudied. We conducted a nationwide, multicentre cross-sectional study using an online questionnaire on a cohort of RT-PCR-confirmed adult COVID-19 patients in Malaysia between 6 June and 30 November 2020. The aim of our study was to investigate their presenting symptoms and assess their chemosensory function using self-ratings of perceived smell, taste, chemesthesis, and nasal blockage. In this cohort of 498 patients, 41.4% reported smell and/or taste loss when diagnosed with COVID-19, which was the commonest symptom. Blocked nose, loss of appetite, and gastrointestinal disturbances were independent predictors of smell and/or taste loss on multivariate analysis. Self-ratings of chemosensory function revealed a reduction in smell, taste, and chemesthesis across the entire cohort of patients that was more profound among those reporting smell and/or taste loss as their presenting symptom. Perceived nasal obstruction accounted for only a small proportion of changes in smell and taste, but not for chemesthesis, supporting viral disruption of sensorineural mechanisms as the dominant aetiology of chemosensory dysfunction. Our study suggests that chemosensory dysfunction in COVID-19 is more widespread than previously reported among Asians and may be related to the infectivity of viral strains.

**Study Registration:** NMRR-20-934-54803 and NCT04390165.

## Introduction

Chemosensory disorders—encompassing smell, taste, and chemesthesis—are increasingly recognised as important symptoms of the Coronavirus Disease 2019 (COVID-19) infection caused by the Severe Acute Respiratory Syndrome Coronavirus 2 (SARS-CoV-2). While early cohort studies reported the most prevalent symptoms to be fever, dry cough, dyspnoea, myalgia, diarrhoea, and sore throat^1,2^, several self-reported symptom studies mainly from the United States, United Kingdom, and Europe have reported smell and taste loss with a prevalence as high as 60–80%^3–7^. The link between COVID-19 and altered chemesthesis—the ability to detect chemically triggered sensations such as spiciness, burning, cooling, or tingling sensation via the trigeminal nerve—was described in a large-scale online survey study by the Parma et al*.* from the Global Consortium for Chemosensory Research (GCCR)^8^.

The extent to which findings from these studies, conducted predominantly in Caucasian populations, can be applied to other populations with differences in genetics, lifestyle, environmental, and cultural factors—as well as differences in infectivity of SARS-CoV-2 genetic variants—remain largely understudied. Understanding the extent of chemosensory disorders within other populations may offer insights into the infectivity of viral strains as well as aid the diagnosis and management of the COVID-19 pandemic within a particular region. A limited number of studies from Asia—derived mainly from health records rather than self-reporting of symptoms—have reported a much lower prevalence of smell and taste loss in COVID-19 (as low as 5%)^9–11^ while no study has reported chemesthesis loss in Asians. A meta-analysis by Von Bartheld et al*.* found a three-fold higher prevalence of smell and taste loss in Caucasians (54.8%) as compared to Asians (17.7%). The difference in prevalence was postulated to be due to geographical differences in the distribution of viral strains harbouring the more infectious D614G spike protein mutation and ethnic-specific differences in genetic variants of viral binding proteins, angiotensin-converting enzymes 2 (ACE-2) and transmembrane protease serine 2 (TMPRSS2)^12,13^.

Here, we report findings from a cross-sectional study that assessed smell, taste, and chemesthesis disturbances in a cohort of COVID-19 patients in Malaysia. Our primary aim was to investigate the timing, severity, qualitative, and quantitative changes of chemosensory function before and during COVID-19. Our secondary aims were to uncover independent predictors of loss of smell and/or taste in COVID-19 and to assess the relationship between changes in smell, taste, chemesthesis, and self-perceived nasal blockage.

## Methods

### Study design

This is a cross-sectional study involving 14 COVID-19 treating public hospitals across all states of Malaysia. A self-administered questionnaire was used to survey patients diagnosed with COVID-19 infection in Malaysia between 6 June and 30 November 2020.

### Patient eligibility

A convenience sample of patients aged ≥ 18 years with COVID-19 infection confirmed with reverse transcription polymerase chain reaction were invited to participate in the survey. Participants were either inpatient or patients who were discharged back to the community at the time of survey administration. Inpatient participants were evaluated to be clinically stable prior to initiating the survey. Those who were in the intensive care unit at the time of study were excluded. An internet link to the questionnaire online was sent to an invited patient after they have given verbal and written informed consent to participate. Phone interviews were conducted to those who did not have internet access or who were illiterate.

### Ethics approval

This study received ethics approval from the Medical Research and Ethics Committee, Ministry of Health, Malaysia (KKM/NIHSEC/P20–1112). All methods in this study were performed in accordance with the relevant guidelines and regulations.

### Questionnaire design

The survey questionnaire was adapted from a pre-existing, validated online questionnaires developed by the GCCR^8^ and the American Academy of Otolaryngology-Head & Neck Surgery (AAO-HNS)^7^. After multiple iterations, a consensus on the final version of the questionnaire was reached among the investigators and satisfied adequate content and face validity per our local setting. Our questionnaire was available in Malay and English, two of the widest spoken languages in Malaysia (Supplementary Materials S1). Participants were asked to report demographic information, symptoms of their COVID-19 diagnosis, time of onset of smell and/or taste loss, severity of symptoms, and whether they had recovered from them at the time of filling the questionnaire. They were given the option to describe any specific changes in smell and taste qualities as a check-all-that applies (CATA) question. They were also asked to quantify their ability to smell, taste, and perceive cooling, tingling and burning sensations (chemesthesis), and perceived nasal obstruction before, during COVID-19, and at the time of filling the questionnaire on a 6-point visual analogue scale (VAS).

### Sample size calculation

Sample size estimation was calculated using the population proportion formula^14^. Prior data indicate that the prevalence of COVID-19-associated chemosensory dysfunction was 47.4%^12^. With a Type I error probability of 5%, precision of 5%, and an estimated prevalence of 50%, we will need to study 384 samples. With an additional 20% dropout rate, the sample size needed was 480 samples.

### Statistical analyses

Associations between categorical variables were tested using Chi-Square test, while differences in mean age were tested using an independent sample *t*-test. Variables associated with smell and taste disturbances were first tested using univariate analysis, and statistically significant variables were then tested in a multivariate logistic regression analysis. The relationship between the categories of symptom severity and the presence of hyposmia or anosmia was tested using Chi-squared test with post hoc Bonferroni correction. The relationship between the categories of symptom severity and self-ratings during COVID-19 diagnosis was tested using Spearman’s rank correlation analysis. Self-ratings of smell, taste, chemesthesis, and nasal congestion before and during COVID-19 diagnosis were tested with Wilcoxon matched pairs signed-rank test. A level of p < 0.05 was considered statistically significant. All statistical analyses were performed using either GraphPad PRISM 9.0 (GraphPad Software Inc., San Diego, CA, USA) or IBM SPSS Statistics (SPSS) version 27.0 (IBM Corporation, Armonk, NY, USA). Principal component analysis (PCA) of the changes in self-ratings of smell, taste, chemesthesis, and nasal blockage [(rating during COVID-19 diagnosis) minus (rating before COVID-19 diagnosis)] was performed in the same manner as previously reported by Parma et al.^8^ using the *prcomp* function from the R default statistics package. PCA is an algorithm that reduces the dimensionality of a dataset while retaining most of the variation in the dataset by identifying directions—termed principal components—along which variation of the data is maximal. Results of the PCA were plotted using functions from the FactoMineR package^15^.

## Results

### Patient recruitment and characteristics

A sample of 827 eligible patients were invited to complete the questionnaire, 743 agreed to participate, and 532 responses were received (response rate 64.3%). 34 responses were excluded due to either not meeting the inclusion criteria, duplicate responses, or inconsistent responses, while the remaining 498 responses were analysed.

Overall, the age of the patients ranged from 18 to 87 (median ± interquartile range [IQR]: 36 ± 24.25 years old). There were 279 males (56%) and 219 (44%) females. The largest ethnic group in our cohort were Malays (76.7%), followed by Chinese (10.0%) and Indians (1.8%), while the remaining (11.5%) included several ethnic groups native to East Malaysia (e.g., Kadazan, Dusun, and Murut) and foreign nationals (3%). 54.4% of patients had at least one comorbidity, the most common being hypertension (17.3%) and diabetes mellitus (13.9%) (Table 1).

**Table 1:** Demographic and clinical characteristics of COVID-19 patients assessed for olfactory & taste disturbances.

Characteristics	Total (N = 498)	Proportion (%)
**Age, years**		
Median (IQR)	36 (28–52)	
**Sex**		
Female	219	43.9
Male	279	56.0
**Ethnicity**		
Malay	382	76.7
Chinese	50	9.8
Indian	9	1.8
Other Malaysian ethnics	47	9.4
Other nationalities	10	2.0
**Pre-existing comorbidities**		
Hypertension	86	17.3
Diabetes	69	13.9
Smoker	56	11.2
Allergies/allergic rhinitis	35	7.0
Obstructive sleep apnoea	28	5.6
Rhinosinusitis	27	5.4
Chronic lung disease/asthma	24	4.8
Obesity	23	4.6
Cardiac disease	13	2.6
Psychiatric disorders	7	1.4
Dyslipidaemia	5	1.0
Previous sinonasal surgery	5	1.0
History of head trauma	4	0.8
Previous head/brain surgery	4	0.8
None	227	45.6
**Presenting symptoms**		
Loss of smell &/or taste	206	41.4
Loss of smell	182	36.6
Loss of taste	169	33.9
Fever	200	40.2
Cough	152	30.5
Sore throat	127	25.5
Malaise	119	23.9
Loss of appetite	108	21.7
Muscle ache	75	15.1
Headache	66	13.3
Nasal congestion	61	12.2
Shortness of breath	57	11.5
Rhinorrhoea	34	6.8
None	138	27.7

### Prevalence, timing, and severity of smell and taste disorders

At time of COVID-19 testing, 206 patients (41.4%) reported either one of loss of smell and/or taste. Among them, 29.3% reported loss of both smell and taste, 7.4% reported loss of smell but not taste, and 5.0% reported loss of taste but not smell. Loss of smell and/or taste was the most common symptom besides fever (40.2%), ahead of cough (30.5%) and sore throat (25.5%) (Table 1). 34.6% of patients with smell loss reported experiencing this before other symptoms whereas 30.7% of patients with taste loss experienced this before other symptoms (Table 2). 7.7% and 3.0% of patients with smell and taste loss respectively experienced this as their only symptom.

**Table 2:** Characteristics of olfactory and taste disturbances in COVID-19 patients.

Characteristics	No	Proportion (%)
Loss of smell^A^	182	36.5
Loss of taste	169	33.9
Loss of smell and taste	145	29.1
Loss of smell and/or taste	206	41.4
Loss of smell without loss of taste	37	7.4
Loss of taste without loss of smell	24	4.8
**Timing of loss of smell**^**B**^		
First symptom	63	34.6
Same time as other symptoms	49	26.9
After other symptoms	56	30.7
Only symptom	14	7.7
**Timing of loss of taste**^**C**^		
First symptom	52	30.7
Same time as other symptoms	47	27.8
After other symptoms	65	38.4
Only symptom	5	3
**Decreased sense of smell 2 weeks before diagnosis**		^B^
No problem	83	45.6
Very mild problem	20	11
Mild or slight problem	38	20.9
Moderate problem	19	10.4
Severe problem	10	5.5
Problem is as bad as it can be	12	6.6
**Decreased sense of taste 2 weeks before diagnosis**		^C^
No problem	78	46.1
Very mild problem	21	12.4
Mild or slight problem	35	20.7
Moderate problem	18	10.7
Severe problem	9	5.3
Problem is as bad as it can be	8	4.7
**Type of smell disturbance**^**B**^		
Anosmia	73	40.1
Hyposmia	100	55.5
Parosmia	28	15.3
Cacosmia	13	7.1
Phantosmia	19	10.4
Fluctuating sense of smell	17	9.3
**Type of taste disturbance**^**C**^		
Sweet	83	49.1
Salty	90	53.2
Sour	75	44.4
Bitter	72	42.6
Umami	48	28.4

^A^Calculated as a proportion of total number of patients (N = 498).

^B^Calculated as a proportion of total number of patients with loss of smell (n = 182).

^C^Calculated as a proportion of total number of patients with loss of taste (n = 169).

In terms of symptom severity, 12.1% and 10.0% of patients with smell and taste loss respectively described their symptoms as “severe” to “as bad as it can be” in the preceding two weeks prior to diagnosis (Table 2). In the CATA question on the type of smell disorders, 73 patients (40.1%) reported complete loss of smell (anosmia) whereas 100 patients (55.5%) reported partial loss of smell (hyposmia). Of note, 17 patients (9.3%) reported fluctuating sense of smell (Table 2). There was no significant correlation between the six categories of symptom severity and the presence of anosmia or hyposmia, as determined by Chi-square test with post hoc Bonferroni correction [Z critical value = 2.86, adjusted alpha level = 0.004 (0.05/12)]. In addition, there was no correlation between these categories of symptom severity and changes in rating scores of the patients when diagnosed with COVID-19 [Spearman’s rank correlation coefficient, r = 0.05, p = 0.5].

### Factors predictive of smell and taste disorders

Loss of smell and/or taste were significantly associated with younger age group (< 50 years), female sex, and the presence of several other symptoms listed in Table 3 on univariate analyses. A multivariate logistic regression analysis was performed using these variables and found that the presence of blocked nose (p < 0.0001, OR 4.95, CI 2.41–10.15), loss of appetite (p < 0.0001, OR 4.16, CI 2.35–7.38), and gastrointestinal disturbances (p = 0.038, OR 2.17, CI 1.04–4.53) were independent predictors of loss of smell and/or taste (Table 3).

**Table 3:** Factors associated with smell and/or taste disturbances among COVID-19 patients in Malaysia.

Variables	Smell &/or taste disturbance		Univariate analysis		Multivariate analysis	
	Present, n (%)	Absent, n (%)	Odds ratio (95% CI)	*p*-value^A^	Odds ratio (95% CI)	*p*-value^B^
**Age group (years)**						
≤ 50	163 (45.0)	199 (55.0)	1.77 (1.17–2.72)	**0.007**	1.30 (0.82–2.08)	0.27
> 50	43 (31.6)	93 (68.4)	1.00 (reference)		1.00 (reference)	
**Sex**						
Male	103 (36.9)	176 (63.1)	0.6591 (0.46–0.94)	**0.02**	0.71 (0.47–1.07)	0.10
Female	103 (47.0)	116 (53.0)	1.00 (reference)		1.00 (reference)	
**Ethnicity**						
Malay	166 (43.5)	216 (56.5)	1.46 (0.95–2.26)	0.09	–	–
Chinese	20 (40.0)	30 (60.0)	0.94 (0.52–1.69)	0.84	–	–
Indian	5 (55.6)	4 (44.4)	1.94 (0.58–6.37)	0.32	–	–
Other Malaysian ethnics	15 (31.9)	32 (68.1)	0.59 (0.31–1.13)	0.10	–	–
**Comorbidities**						
Hypertension	32 (37.2)	54 (62.8)	0.81 (0.51–1.29)	0.40	–	–
Diabetes	25 (36.2)	44 (63.8)	0.78 (0.46–1.31)	0.43	–	–
Smoking	21 (37.5)	35 (62.5)	0.83 (0.48–1.48)	0.57	–	–
Allergies/allergic rhinitis	16 (45.7)	19 (54.3)	1.21 (0.63–2.41)	0.60	–	–
Obstructive sleep apnoea	14 (50.0)	14 (50.0)	1.45 (0.68–3.05)	0.43	–	–
Rhinosinusitis	12 (44.4)	15 (55.6)	1.16 (0.53–2.59)	0.84	–	–
Chronic lung disease/asthma	14 (53.9)	12 (46.2)	1.70 (0.80–3.78)	0.22	–	–
Obesity	12 (52.2)	11 (47.8)	1.41 (0.59–3.10)	0.52	–	–
Cardiac disease	6 (46.2)	7 (53.9)	1.22 (0.40–3.34)	0.78	–	–
Psychiatric disorders	3 (42.8)	4 (57.1)	1.06 (0.27–4.00)	> 0.99	–	–
Previous sinonasal surgery	3 (42.8)	4 (57.1)	1.06 (0.27–4.00)	> 0.99	–	–
History of head trauma	1 (25.0)	3 (75.0)	1.66 (0.59–9.12)	0.65	–	–
Previous head/brain surgery	1 (20.0)	4 (80.0)	0.35 (0.03–2.14)	0.65	–	–
**Associated symptoms**						
Fever	110 (55)	90 (45)	2.53 (1.78–3.69)	** < 0.001**	1.47 (0.92–2.348)	0.11
Cough	74 (48.7)	78 (51.3)	1.54 (1.03–1.57)	**0.03**	0.64 (0.39–1.05)	0.08
Sore throat	74 (58.3)	53 (41.7)	2.53 (1.67–3.80)	** < 0.001**	1.53 (0.93–2.54)	0.10
Malaise	72 (60.5)	47 (39.5)	2.80 (1.81–4.24)	** < 0.001**	0.93 (0.52–1.68)	0.81
Loss of appetite	79 (73.8)	28 (26.2)	5.87 (3.64–9.59)	** < 0.001**	4.17 (2.35–7.38)	** < 0.001**
Muscle ache	43 (57.3)	32 (42.7)	2.14 (1.29–3.55)	**0.003**	0.71 (0.36–1.39)	0.32
Headache	46 (69.7)	20 (30.3)	3.91 (2.21–6.71)	** < 0.001**	1.40 (0.68–2.89)	0.36
Nasal congestion	46 (76.7)	14 (23.3)	5.71 (3.02–10.37)	** < 0.001**	4.95 (2.41–10.15)	** < 0.001**
Rhinorrhoea	22 (64.7)	12 (35.3)	2.79 (1.36–5.64)	**0.004**	1.24 (0.52–2.97)	0.63
Chills	20 (64.5)	11 (35.5)	2.75 (1.26–6.04)	**0.007**	1.48 (0.60–3.69)	0.40
Gastrointestinal disturbances	44 (73.3)	16 (26.7)	4.69 (2.56–8.52)	** < 0.001**	2.17 (1.04–4.53)	**0.04**

Bold values indicate statistical significance (p < 0.05).

(–) not included in multivariate model.

^A^p-values were calculated using Chi square test (or Fisher’s exact test when n < 5 in any cell).

^B^p-values for multivariate analysis were calculated using multiple logistic regression (Forward method).

### Quantitative changes of smell, taste, chemesthesis, and nasal obstruction during COVID-19

**Figure 1:**
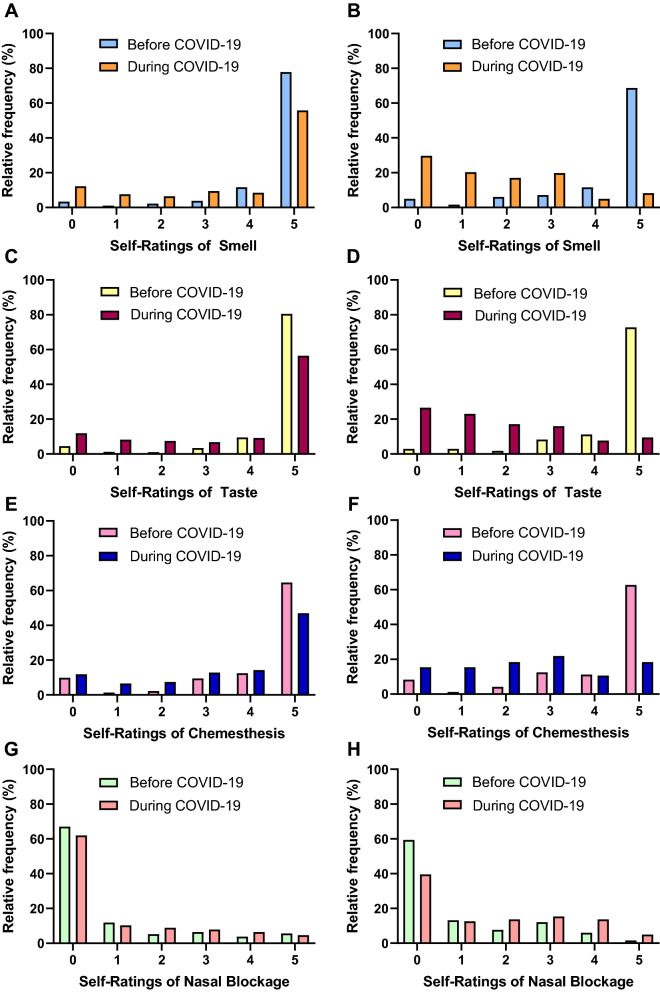
Self-ratings of smell, taste, chemesthesis, and nasal congestion before and during COVID-19. Interleaved histograms represent self-ratings for smell, taste, chemesthesis, and nasal congestion on a 6-point visual analogue scale before and during COVID-19 in all patients (**A**,**C**,**E,G**), and the subset of patients who report changes in smell (**B**,**H**) and taste (**D**,**F**).

The distribution of patients’ self-ratings of smell, taste, and chemesthesis, and nasal obstruction before and during COVID-19 diagnosis are depicted in Fig. 1. There were statistically significant changes in self-ratings of smell, taste, chemesthesis, and nasal blockage in the total patient cohort and the subgroups before and during COVID-19 diagnosis as measured by Wilcoxon matched pairs signed-rank test (Table 4).

**Table 4:** Self-ratings of smell, taste, chemesthesis, and nasal obstruction before and during COVID-19.

Variable	Before COVID-19		During COVID-19		Ratings below/above cut-off score (%) ^A^		p-value^B^
	Mean	SE	Mean	SE	Before COVID-19	During COVID-19	
**Smell ratings**							
All patients	4.53	0.05	3.62	0.08	10.4	35.6	< 0.001
Smell disturbance only	4.25	0.10	1.74	0.12	19.6	86.7	< 0.001
**Taste ratings**							
All patients	4.54	0.05	3.63	0.08	10.0	34.2	< 0.001
Taste disturbance only	4.40	0.09	1.83	0.12	16.1	82.9	< 0.001
**Chemesthesis ratings**							
All patients	4.07	0.07	3.52	0.08	22.8	38.7	< 0.001
Taste disturbance only	4.05	0.12	2.52	0.13	26.0	71.1	< 0.001
**Nasal blockage ratings**							
All patients	0.85	0.07	1.00	0.07	21.0	27.7	< 0.01
Smell disturbance only	0.97	0.10	1.66	0.12	27.4	47.8	< 0.001

^A^For smell, taste and chemesthesis, ratings are below the cut-off score of 4. For nasal blockage, ratings are above the cut-off score of 1.

^B^Wilcoxon matched pairs signed-rank test.

We observed an increase in the percentage of patients with smell, taste, and chemesthesis ratings lower than a cut-off point of 4 compared to their baseline ratings prior to COVID-19 diagnosis (smell: 35.6% from 10.4%; taste: 34.2% from 10.0%; chemesthesis: 38.7% from 22.8%) (Fig. 1). Subgroup analysis of only those who reported smell loss as their presenting complaint (n = 182) revealed a higher increase in the proportion of smell ratings lower than 4 from 19.6% to 86.7%. Similarly, those who reported taste loss as their presenting complaint (n = 169) had a greater increase in the proportion of taste and chemesthesis ratings below 4 (taste, 82.9% from 16.1%; chemesthesis: 71.1% from 26%).

In parallel, we observed a slight increase in perceived nasal obstruction related to COVID-19. At baseline, 21% of patients reported a nasal blockage rating of greater than 1, which increased to 27.7% when diagnosed with COVID-19. Subgroup analysis of only patients who reported smell loss as their presenting symptom (n = 182) found 47.8% reporting a nasal blockage rating of greater than 1, from 27.4% at baseline. This observation is concordant with findings of significant association of smell loss with nasal congestion on multivariate analysis.

### Relationship between self-ratings of smell, taste, chemesthesis, and nasal obstruction

**Figure 2:**
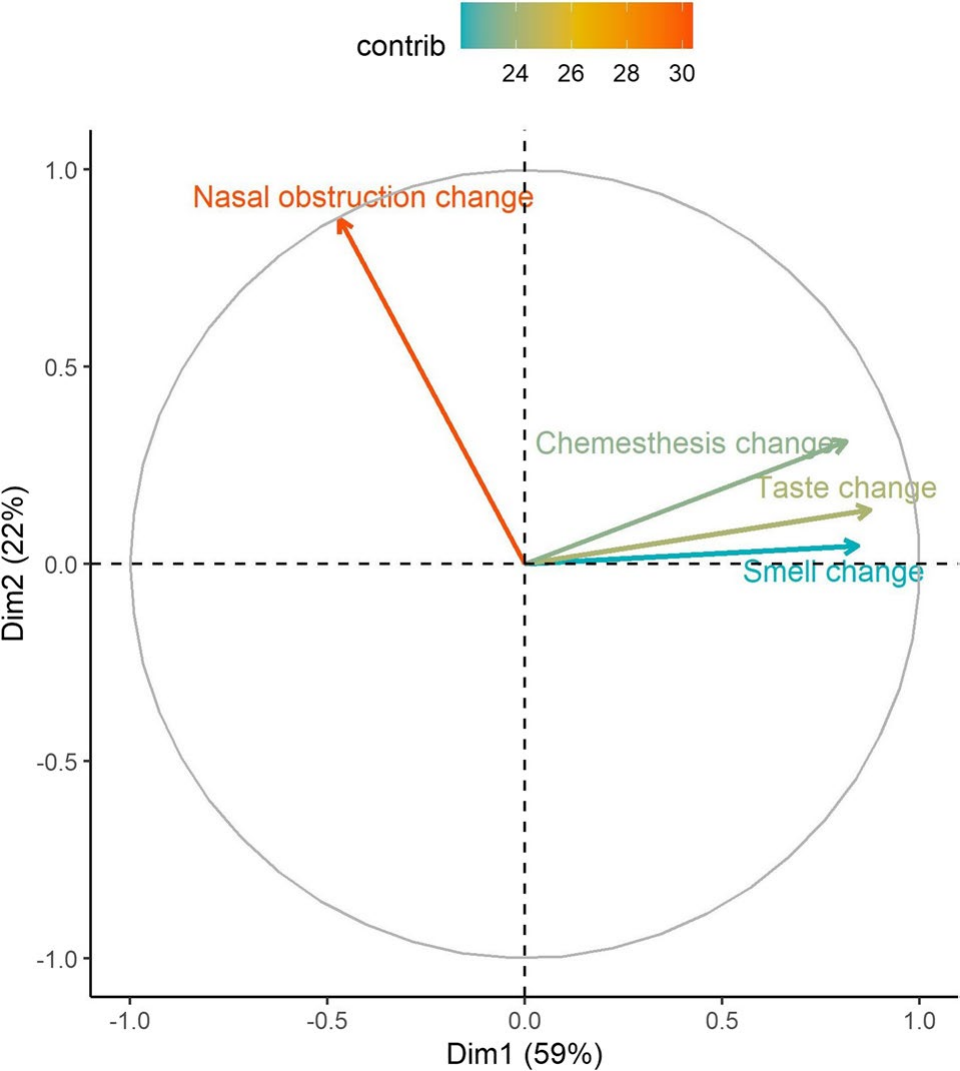
Principal component analysis (PCA) of difference scores of ratings of smell, taste, and chemesthesis [(rating during COVID-19 diagnosis) minus (rating before COVID-19 diagnosis)]. The findings of the PCA are depicted by a correlation circle of vectors representing changes in perceived smell, taste, chemesthesis and nasal blockage with the first (Dimension 1, abscissa) and second (Dimension 2, ordinate) principal components.

To further characterise the relationship between changes in perceived nasal obstruction and changes in the three chemosensory modalities, we performed a principal component analysis of the changes in self-ratings of smell, taste, chemesthesis, and perceived nasal blockage (during minus before diagnosis of COVID-19) (Fig. 2). This analytic approach was previously employed by Parma et al*.*^8^ to determine whether changes in chemosensory function can be attributed to nasal obstruction. It leverages the orthogonal features of these principal components to evaluate the degree of statistical dependence between changes in chemosensory ability and perceived nasal obstruction. In our analysis, the two orthogonal principal components, Components 1 and 2, accounted for 59% and 22% of the total multidimensional variances respectively. Changes in self-ratings of smell, taste, and chemesthesis clustered together and correlated strongly with Component 1 (smell: r = 0.837, taste: r = 0.871, chemesthesis: r = 0.815), while showing negligible to weak positive correlation with the Component 2 (smell: r = 0.066, taste: 0.097, and chemesthesis: 0.333). In contrast, changes in self-ratings of nasal obstruction demonstrated only a moderate negative correlation with Component 1 (r = − 0.474) but strong positive correlation (r = 0.873) with Component 2. The PCA loading vectors for changes in chemesthesis and nasal obstruction formed a right angle indicating that they were not correlated and statistically independent of each other, whereas vectors for smell and taste changes formed a small obtuse angle with the vector for nasal obstruction, indicating a weak negative correlation. These PCA findings suggest that nasal obstruction could only account for a small proportion of smell and taste changes, but not for chemesthesis.

### Qualitative changes of smell and taste during COVID-19

Qualitative changes in smell were uncommon among those who experienced smell disturbances—only 28 patients (15.3%) experienced parosmia, 19 patients (10.4%) experienced phantosmia, and 13 patients (7.1%) experienced cacosmia. The distinction between parosmia and cacosmia were made on the basis of patients’ selected response to Question 27 of the Questionnaire (Supplementary Material S1). Patients who selected “Smells smell different than they did before (the quality of smell has changed)” were considered to have parosmia, whereas those who selected “Smells smell unpleasant” were considered to have cacosmia.

Among participants who reported gustatory changes, 33 patients (19.5%) reported impairment of a single taste quality and 106 patients (62.7%) reported impairment of 2 or more taste qualities in the CATA question. 30 patients (17.8%) did not respond to this question (Table 2). Salty taste was the most frequently reported change (53.2%) followed by sweet (49.1%), sour (44.4%), bitter (42.6%), and umami/savoury (28.4%) taste.

### Recovery of smell and taste disorders

Among the patients with smell and/or taste disorders, 90.2% (n = 186) of them reported recovery of their symptoms at the time of answering the questionnaire. This was on average 113 ± 31 days (mean ± SD) from the date of their COVID-19 diagnosis. Self-ratings of sense of smell, taste, chemesthesis, and nasal congestion at this time-point returned to pre-COVID-19 baseline levels in all patients and in the subset of patients who reported the chemosensory disorders [Mean smell rating: all patients 4.66 ± 0.86; smell disturbance only 4.49 ± 0.93; mean taste rating: all patients 4.74 ± 0.78; taste disturbance only 4.61 ± 0.82; mean chemesthesis rating: all patients 4.16 ± 1.51; taste disturbance only 4.20 ± 1.36; mean nasal congestion rating: all patients 0.54 ± 1.22; smell disturbance only 0.60 ± 1.23; mean ± SD].

## Discussion

Our survey which involved close to 500 patients treated in 14 COVID-19-treating public hospitals in Malaysia revealed that loss of smell and/or taste was not only an early symptom of COVID-19 infection, but also the commonest symptom in this cohort, involving 40.2% of our patients. In comparison, a previous multicentre nationwide study from Malaysia based on the health records of 5889 hospitalised patients found the most common clinical manifestation of COVID-19 to be cough (32.2%), fever (29.5%), sore throat (14.3%), rhinorrhea (10.3%) and shortness of breath (5.3%), whereas anosmia and ageusia only constituted a small minority of cases (2.8% and 0.7%, respectively)^16^. In addition, a single-centre Malaysian study of the health records of 199 COVID-19 patients reported only 6.25% of cases experiencing anosmia^17^. By comparison, a telephone questionnaire study from another single centre Malaysian study of 145 patients reported the prevalence of olfactory dysfunction and dysgeusia to be 21.4% and 23.4% respectively^18^. The discrepancy between our findings and these previous studies is likely because self-reporting is more sensitive in identifying symptom-based conditions compared to physician-reporting in health records^19–21^. The rarity of patients reporting parosmia and phantosmia in our study was consistent with the findings of Parma et al*.*^8^, although other studies have reported higher prevalence of parosmia and phantosmia^5,22^.

The findings of chemosensory self-ratings revealed that COVID-19 chemosensory loss in our cohort was not only confined to smell and taste but also involved chemesthesis. While the proportion of smell and taste loss in our cohort is higher than that observed in other Asian studies^9–11^, its magnitude and extent is not as marked as that reported by Parma et al*.*^8^ and other studies involving predominantly Caucasian populations^3–7^. Possible reasons for this may include the influence of cultural context and geographical location on the awareness and perception of smell and taste^23–25^. In addition, the D614G mutation of the coronavirus spike protein and ethnic differences in the frequency of variants of the virus-binding entry proteins (ACE-2 and TMPRSS2) have been proposed to facilitate virus entry in the olfactory epithelium and taste buds, thus increasing the likelihood of smell and taste disturbance^12,13^. A recent systematic review and meta-analysis found that South Asian populations infected predominantly with G614 virus had a much higher prevalence of anosmia compared with the same ethnic population infected mostly with the D614 strain, suggesting that D614G mutation is a major contributing factor that increases the prevalence of anosmia in COVID-19^26^. In a study on predominantly South Asian foreign workers with mild or no symptoms at a COVID-19 community isolation facility in Singapore, the prevalence of anosmia and ageusia was 3.0% and 2.6% respectively^27^. Retrospective analysis of publicly-available SARS-CoV-2 genome sampled from this population found a predominance of D614 strain, supporting the hypothesis of D614G mutation-mediated increase in the prevalence of anosmia^28^. While the majority of early cases of COVID-19 in Malaysia between February to April 2020 involved the SARS-CoV-2 lineage B.6 that did not harbour the D614G mutation^29^, the rapid spread of new cases in Malaysia between the months of May to December 2020 was found to be due to an increase in frequency of viral strains harbouring the D614G mutation^30^. Hence, it is plausible that the higher proportion of chemosensory disturbances in our cohort relative to other studies from Asia may also reflect the increasing frequency of viral strains harbouring the D614G mutation during the period of our study, although further work is needed to verify this hypothesis.

The association between loss of appetite and gastrointestinal disturbances with loss of smell or taste on multivariate analysis is congruent with observations from large-scale population studies of COVID-19 symptoms that demonstrated association between anosmia with loss of appetite and gastrointestinal symptoms^6,31,32^. The physiological relevance of these findings is highly plausible since our appetite is tightly linked to smell and taste, and may reflect concomitant SARS-CoV-2 viral infection of the olfactory and gastrointestinal tract epithelia^33,34^.

Our findings from the principal component analysis suggest that factors other than nasal congestion underlie most of the chemosensory changes, and that sensorineural impairment was likely the dominant mechanism in our cohort with only a small proportion of smell and taste loss may be attributed to nasal congestion. Importantly, chemesthesis loss was independent of nasal congestion. Olfactory dysfunction has been proposed to be due to conductive loss from mucosal obstruction of the olfactory cleft^35^ or sensorineural impairment from the direct effect of the virus on olfactory epithelium^36–40^. In particular, SARS-CoV-2 viral infection has been shown to cause anosmia by infecting the non-neuronal sustentacular cells in the olfactory epithelium that express ACE-2, the receptor required for viral entry into the cell^36,37^. Other mechanisms have implicated damage to the olfactory neurons from pro-inflammatory cytokines^39^ and disruption of signalling from olfactory sensory neurons to the olfactory bulb^40^. However, whether loss of neuronal cells actually occurs in COVID-19 and causes anosmia is currently controversial due to a lack of convincing evidence for this ^41^.

Mechanisms for taste loss is less clear, since taste is closely linked to smell and nasal congestion. However, SARS-CoV-2 may infect taste chemoreceptor cells since ACE-2 is expressed on tongue keratinocytes^42,43^ or cranial nerves responsible for gustation (cranial nerves VII, IX and X) although evidence for this is lacking. Loss of chemesthesis have been hypothesised to be due to viral infection of the trigeminal nerve although, again, evidence for this is lacking^8^. Our findings support a dominant role for sensorineural mechanisms in SARS-CoV-2-related loss of smell, taste, and chemesthesis.

The major limitation of our study is the reliance on self-reporting of chemosensory function, which is subjective. Objective assessment of olfactory dysfunction in COVID-19 have been reported using a number of psychophysical tests such as Sniffin’ Sticks test^44^, Connecticut Chemosensory Research Centre orthonasal olfaction test^45^, and the University of Pennsylvania Smell Identification Test^46^. Likewise, gustatory dysfunction in COVID-19 have also been studied using objective tools such as a four-item taste test (sweet, sour, salty, and bitter)^45^ and taste-strips impregnated with four taste qualities^47^. Nonetheless, self-reporting of chemosensory function is still widely used with reasonable accuracy rates between 70 and 80%^48,49^, and may be useful for remote assessment of patients in the setting of a pandemic. Moreover, there is evidence to suggest that objective testing is not always the most sensitive approach in detecting smell and taste loss in COVID-19. Boscutti et al*.* recently published a systematic review and meta-analysis of all observational studies reporting the prevalence and longitudinal trajectories of olfactory and gustatory disorders in COVID-19 using patient self-reporting and objective psychophysical tests^50^. They found higher prevalence from self-reporting compared to psychophysical testing in some studies whereas the opposite was true for other studies, leading them to conclude that psychophysical testing was not always the most sensitive measure^50^. The replicability of tests across different countries has been suggested as a possible confounding factor^50^. Therefore, while objective tests for smell and taste are important, there is value in studying the chemosensory disorder in COVID-19 using self-reporting. Other limitations of our study include recall bias, the use of convenient sampling, and the lack of validation of the translated version of the questionnaire within our Malaysian population.

We accounted for individual differences in baseline chemosensory abilities and the use of rating scales in two ways—first, our study used a within-subject design where the participants rated their abilities at different time points (before and during COVID-19). The same individual participates in all conditions, hence controlling for differences in participant characteristics. Second, we analysed the differences in ratings between the two time-points (“during COVID-19” minus “before COVID-19”), instead of using absolute ratings. Hence, this approach precludes the need to normalise ratings to the baseline since we are not analysing the absolute values. Of note, this study design and method of analysis have been previously employed in large scale studies of chemosensory loss in COVID-19 using self-ratings^8,51,52^.

Our study also unveils opportunities to improve our understanding of COVID-19-associated chemosensory disturbances in Asian versus Caucasian populations. Future studies should compare these self-reported findings to culturally-adapted smell identification tests, such as a recently developed Malaysian version of Sniffin’ Stick Smell Identification test^53^, psychophysical tests of smell and taste, and imaging to assess the patency of the olfactory clefts and nasal cavity. Recent loss of smell has been suggested to be the best predictor of COVID-19 diagnosis^51^, and therefore, it would be of significant clinical importance to determine whether or not this is the case in Asian populations.

## Conclusion

In summary, our study reveals widespread loss of smell, taste, and chemesthesis in Malaysian COVID-19 patients that manifested as early symptoms of infection. These chemosensory losses largely cannot be accounted for by nasal blockage, suggesting a predominantly sensorineural aetiology. These findings challenge earlier reports that smell and taste loss in COVID-19 are less common among Asians, suggesting that these symptoms may be more common than previously thought and may be related to the infectivity of the SARS-CoV-2 strains.

## Supplementary Information

Supplementary Information.

## Data Availability

The datasets generated during and/or analysed during the current study are available from the corresponding author on reasonable request.
