# Supplementary material for: Self-reported symptom study of COVID-19 chemosensory dysfunction in Malaysia
Source: Sci Rep. 2022 Feb 8;12:2111. doi: 10.1038/s41598-022-06029-6 (PMC8826975; doi:10.1038/s41598-022-06029-6)
Supplement: Supplementary file 1 — Supplementary Information. [file 41598_2022_6029_MOESM1_ESM.docx]

**SUPPLEMENTARY MATERIALS**

**Study Questionnaire (English Version)**

**1. Age:**

__________________

**2. Gender:**

- Male
- Female

**3. Nationality:**

- Malaysian
- Non-Malaysian

**4. Ethnicity:**

- Malay
- Chinese
- Indian
- Iban
- Bidayuh
- Melanau
- Kadazan
- Dusun
- Murut
- Others: _________

**5. Occupation:**

____________________

**6. Which district/town/city do you live in?**

**____________________**

**7. Which state do you live in?**

**_____________________**

**8. Have you been diagnosed with COVID-19?**

- Yes
- No

**9. When were you diagnosed with COVID-19 (Date of 1^st^ RT-PCR test positive result)?**

Date: ___________________ (estimate date if unable to recall)

**10. Is the source of your COVID-19 infection identifiable?**

- Yes
- No

**11. Were you diagnosed with any OTHER respiratory illness (not COVID-19) in the last 2 weeks before your diagnosis with COVID-19?**

- Bacterial throat infection
- Flu (Influenza)
- Another viral illness
- Others: ________
- None

**12. Please state your risk factors for COVID-19 infection (*select as many as applicable*):**

- None
- Healthcare worker (Doctor, Dentist, Nurse, Pharmacist, Allied Health Professionals)
- Non-clinical Hospital Staff (including cleaners)
- First Responder (Police, Firefighter, Military, Pre-Hospital Care Staff)
- Close contact with a confirmed case
- Travel/resided in foreign countries/areas with widespread community transmission within 14 days before onset of symptoms
- Mass gatherings (e.g., *Jemaah Tabligh* at Sri Petaling Mosque)
- Living in crowded conditions (Dormitories, Nursing Home, Prison, Shelters)
- Homelessness
- Others, please state: ___________

**13. Please choose any of the following health conditions that you have/conditions that apply to you (*select as many as applicable*):**

- None
- Smoking
- History of head trauma (e.g. from road traffic accidents)
- Previous surgery to the head/brain
- Previous sinus/nose surgery
- Rhinosinusitis
- Allergies/Allergic rhinitis
- Chronic respiratory disease/asthma
- Cardiac disease
- Hypertension
- Diabetes Mellitus
- Neurologic disease (e.g., Parkinson’s, Alzheimer’s)
- Psychiatric disorders (e.g., Depression, Schizophrenia)
- Obesity
- Obstructive Sleep Apnoea (OSA)
- Others, please state: ________

**14. What symptoms did you have when you were diagnosed with COVID-19? (*Please select as many as applicable*)**

- None
- Fever
- Chills
- Malaise
- Loss of smell
- Changes in food flavour/taste disturbance
- Cough
- Sore throat
- Shortness of breath
- Headache
- Nasal congestion/blockage
- Rhinorrhoea (Runny nose)
- Loss of appetite
- Muscle aches
- Gastrointestinal disorder (e.g., abdominal pain/diarrhoea/nausea)
- Ear symptoms (e.g., ear discharge/ear pain/tinnitus/reduced hearing)
- Eye symptoms (e.g., red eyes/watery eyes/eye swelling)
- Other: _________

**15. Did you experience any loss of smell when you were diagnosed with COVID-19?**

- Yes
- No

**16. If you had any loss of smell, was this your first symptom?**

- YES, loss of smell was my first symptom
- NO, loss of smell was not my first symptom
- Loss of smell was my only symptoms
- I do not have any loss of smell

**17. If loss of smell was NOT your first symptom, when did this symptom begin?**

- At the same time as the other COVID-19 symptoms (as those in Question 14
- After the other COVID-19 symptoms (as those in Question 14)
- Loss of smell is my only symptom
- I do not have any loss of smell

**18. Did you experience any taste disturbance?**

- Yes
- No

**19. If you had any taste disturbance, was this your first symptom?**

- YES, taste disturbance was my first symptom
- NO, taste disturbance was not my first symptom
- Taste disturbance was my ONLY symptom
- I do not have taste disturbance

**20. If taste disturbance was not your first symptom, when did this symptom begin?**

- At the same time as the other COVID-19 symptoms (as those in Question 14
- After the other COVID-19 symptoms (as those in Question 14)
- Taste disturbance was my only symptom
- I do not have taste disturbance

**21. Did your COVID-19 infection worsen or improve after you started to notice your smell/taste disturbance?**

- Worsen
- Improve

**22. Now, have you recovered from your smell/taste disturbance?**

- Yes
- No

**23. Did you receive any specific treatment for your taste/smell disorder?**

- Yes
- No

**24. Please rate these problems as they have been over the past 2 weeks BEFORE your diagnosis with COVID-19 (Circle where appropriate, choose 0 if you did not experience this problem/symptom)**

| **No.** | **Problem** | **No Problem** | **Very Mild Problem** | **Mild or Slight Problem** | **Moderate Problem** | **Severe Problem** | **Problem as bad as it can be** |
| --- | --- | --- | --- | --- | --- | --- | --- |
| 1 | Decreased sense of smell | 0 | 1 | 2 | 3 | 4 | 5 |
| 2 | Decreased sense of taste | 0 | 1 | 2 | 3 | 4 | 5 |
| 3 | Ear Pain | 0 | 1 | 2 | 3 | 4 | 5 |
| 4 | Ear Fullness | 0 | 1 | 2 | 3 | 4 | 5 |

**Please tick as appropriate**

**25. Rate your ability to smell BEFORE your COVID-19 diagnosis?**

| No sense of smell |  |  |  |  |  |  |  |  |  | Excellent sense of smell |
| --- | --- | --- | --- | --- | --- | --- | --- | --- | --- | --- |
| 🄋 |  | ➀ |  | ➁ |  | ➂ |  | ➃ |  | ➄ |

**26. Rate your ability to smell DURING your COVID-19 diagnosis?**

| No sense of smell |  |  |  |  |  |  |  |  |  | Excellent sense of smell |
| --- | --- | --- | --- | --- | --- | --- | --- | --- | --- | --- |
| 🄋 |  | ➀ |  | ➁ |  | ➂ |  | ➃ |  | ➄ |

**27. Have you experienced any of the following changes in smell with your COVID-19 diagnosis?** (select as many as applicable)

- I cannot smell at all
- Smells smell less strong than they did before
- Smells smell different than they did before (the quality of smell has changed)
- Smells smell unpleasant
- I can smell things that aren’t there (e.g. I smell burning when nothing is on fire)
- Sense of smell fluctuates (comes and goes)
- No change in sense of smell

**28. Please describe any additional changes in smell:**

_________________________

**29. Rate your ability to smell NOW**

| No sense of smell |  |  |  |  |  |  |  |  |  | Excellent sense of smell |
| --- | --- | --- | --- | --- | --- | --- | --- | --- | --- | --- |
| 🄋 |  | ➀ |  | ➁ |  | ➂ |  | ➃ |  | ➄ |

**30. How blocked was your nose BEFORE your diagnosis of COVID-19?**

| Not at all blocked |  |  | |  | |  | |  | |  | |  | |  | |  | | Completely blocked | |
| --- | --- | --- | --- | --- | --- | --- | --- | --- | --- | --- | --- | --- | --- | --- | --- | --- | --- | --- | --- |
| 🄋 |  | | ➀ | |  | | ➁ | |  | | ➂ | |  | | ➃ | |  | | ➄ |

**31. How blocked was your nose DURING your diagnosis of COVID-19?**

| Not at all blocked |  |  | |  | |  | |  | |  | |  | |  | |  | |  | Completely blocked | | |
| --- | --- | --- | --- | --- | --- | --- | --- | --- | --- | --- | --- | --- | --- | --- | --- | --- | --- | --- | --- | --- | --- |
| 🄋 |  | | ➀ | |  | | ➁ | |  | | ➂ | |  | | ➃ | |  | | | ➄ |  |

**32. How blocked is your nose NOW?**

| Not at all blocked |  |  | |  | |  | |  | |  | |  | |  | |  | | Completely blocked | |
| --- | --- | --- | --- | --- | --- | --- | --- | --- | --- | --- | --- | --- | --- | --- | --- | --- | --- | --- | --- |
| 🄋 |  | | ➀ | |  | | ➁ | |  | | ➂ | |  | | ➃ | |  | | ➄ |

**33. Rate your ability to taste BEFORE your diagnosis of COVID-19?**

| No sense of taste |  |  |  |  |  |  |  |  |  | Excellent  sense of taste |
| --- | --- | --- | --- | --- | --- | --- | --- | --- | --- | --- |
| 🄋 |  | ➀ |  | ➁ |  | ➂ |  | ➃ |  | ➄ |

**34. Rate your ability to taste DURING your diagnosis of COVID-19?**

| No sense of taste |  |  |  |  |  |  |  |  |  | Excellent sense of taste |
| --- | --- | --- | --- | --- | --- | --- | --- | --- | --- | --- |
| 🄋 |  | ➀ |  | ➁ |  | ➂ |  | ➃ |  | ➄ |

**35. Have you experienced any changes to specific tastes with your recent COVID-19 diagnosis?** (select as many as applicable)

- Sweet
- Salty
- Sour
- Bitter
- Savoury/Umami
- No change to sense of taste

**36. Rate your ability to taste NOW**

| No sense of taste |  |  |  |  |  |  |  |  |  | Excellent sense of taste |
| --- | --- | --- | --- | --- | --- | --- | --- | --- | --- | --- |
| 🄋 |  | ➀ |  | ➁ |  | ➂ |  | ➃ |  | ➄ |

**The following THREE questions related to other sensations in your mouth like burning, cooling or tingling. For example, chilli, mint, or carbonated drinks**

**37. Rate your ability to feel these other sensations BEFORE your COVID19 diagnosis?**

| Not sensitive at all |  |  |  |  |  |  |  |  |  | Very  Sensitive |
| --- | --- | --- | --- | --- | --- | --- | --- | --- | --- | --- |
| 🄋 |  | ➀ |  | ➁ |  | ➂ |  | ➃ |  | ➄ |

**38. Rate your ability to feel these other sensations BEFORE your COVID19 diagnosis?**

| Not sensitive at all | |  | |  | |  | |  | |  | |  | |  | |  | |  | Very  Sensitive |
| --- | --- | --- | --- | --- | --- | --- | --- | --- | --- | --- | --- | --- | --- | --- | --- | --- | --- | --- | --- |
| 🄋 |  | | ➀ | |  | | ➁ | |  | | ➂ | |  | | ➃ | |  | | ➄ |

**39. Rate your ability to feel these other sensations NOW**

| Not sensitive at all | |  | |  | |  | |  | |  | |  | |  | |  | |  | Very  Sensitive |
| --- | --- | --- | --- | --- | --- | --- | --- | --- | --- | --- | --- | --- | --- | --- | --- | --- | --- | --- | --- |
| 🄋 |  | | ➀ | |  | | ➁ | |  | | ➂ | |  | | ➃ | |  | | ➄ |

**40. What ear symptom(s) did you experience when you were diagnosed with COVID-19?** (*Please select as many as applicable)*

- None
- Ear pain/tenderness
- Ear discharge
- Reduced hearing/loss of hearing
- Ringing sound in the ears (tinnitus)
- Spinning sensation (vertigo)
- Others, please state: __________________

**41. Did your ear symptom(s) resolve?**

- Yes
- No
- I did not have ear symptom

**42. Did you receive specific any treatment for your ear symptom(s):**

- Yes
- No
- I did not have ear symptom

**43. What is your current COVID-19 infection status?**

- Active – still admitted in hospital
- Discharged home

**44. Comments/Additional Information:**

_________________________________________________________________________

_________________________________________________________________________

**If you agree to be contacted, please provide us your contact details:** (optional)

Telephone number (Mobile): ______________________________________________________

Telephone number (Home): _______________________________________________________

E-mail: _______________________________________

**How would you prefer to be contacted?** (*Please select as many as applicable*)

- Telephone
- WhatsApp
- Email

**This form is filled up by:**

- Myself (participant)
- Proxy – family member/caretaker
- Proxy – treating team

**Mobile phone number of proxy** (optional/if applicable): _______________________

**Thank you very much for your participation in this survey!**
